# Noradrenergic Modulation of Fear Conditioning and Extinction

**DOI:** 10.3389/fnbeh.2018.00043

**Published:** 2018-03-13

**Authors:** Thomas F. Giustino, Stephen Maren

**Affiliations:** Department of Psychological and Brain Sciences, Texas A&M Institute for Neuroscience, Texas A&M University, College Station, TX, United States

**Keywords:** fear, extinction, consolidation, locus coeruleus, norepinephrine

## Abstract

The locus coeruleus norepinephrine (LC-NE) system plays a broad role in learning and memory. Here we begin with an overview of the LC-NE system. We then consider how both direct and indirect manipulations of the LC-NE system affect cued and contextual aversive learning and memory. We propose that NE dynamically modulates Pavlovian conditioning and extinction, either promoting or impairing learning aversive processes under different levels of behavioral arousal. We suggest that under high levels of stress (e.g., during/soon after fear conditioning) the locus coeruleus (LC) promotes cued fear learning by enhancing amygdala function while simultaneously blunting prefrontal function. Under low levels of arousal, the LC promotes PFC function to promote downstream inhibition of the amygdala and foster the extinction of cued fear. Thus, LC-NE action on the medial prefrontal cortex (mPFC) might be described by an inverted-U function such that it can either enhance or hinder learning depending on arousal states. In addition, LC-NE seems to be particularly important for the acquisition, consolidation and extinction of contextual fear memories. This may be due to dense adrenoceptor expression in the hippocampus (HPC) which encodes contextual information, and the ability of NE to regulate long-term potentiation (LTP). Moreover, recent work reveals that the diversity of LC-NE functions in aversive learning and memory are mediated by functionally heterogeneous populations of LC neurons that are defined by their projection targets. Hence, LC-NE function in learning and memory is determined by projection-specific neuromodulation that accompanies various states of behavioral arousal.

## Introduction

The locus coeruleus norepinephrine (LC-NE) system has numerous functions including regulating the sleep-wake cycle, arousal, respiration, motivation, cognition, and learning and memory. In particular, NE plays a broad role in the formation and retrieval of emotional memories. As such, NE is a candidate molecule for the treatment of trauma- and stressor-related disorders and a number of studies suggest that the NE system may be dysregulated in posttraumatic stress disorder (PTSD; Yehuda et al., 1992; Bremner et al., 1996; Southwick et al., 1997, 1999a,b,c; Morilak et al., 2005; Arnsten, 2009, 2015; Rodrigues et al., 2009; Arnsten et al., 2015; Raio and Phelps, 2015; Giustino et al., 2016b; Kroes et al., 2016a).

Trauma- and stressor-related disorders, including PTSD, are pervasive in our society, making improved treatment options an important issue for researchers and clinicians alike. Although most individuals will experience or witness a traumatic event during their lifetime, only of portion of those will go on to develop such disorders. PTSD has a life time prevalence of approximately 10% in the general population (Kessler et al., 1995, 2005) and this number only increases for members of the military (Koenen et al., 2008; Pitman et al., 2012). Research has attempted to understand the underlying pathophysiology of these disorders with an aim toward empirically driven therapeutic approaches. However, most approaches, whether it is cognitive behavioral therapies (such as exposure therapy), pharmaceutical agents, or a combination of the two, fall short in treating these debilitating disorders (Tawa and Murphy, 2013). Despite the extensive research on this topic, the use of NE-altering drugs for PTSD treatment remains controversial.

In the laboratory, Pavlovian fear conditioning procedures are used to understand the neurobiology of aversive learning and memory. In a typical paradigm, a neutral cue (conditioned stimulus, CS, such as an auditory tone) is paired with an aversive unconditioned stimulus (US, such as a mild electric footshock). Rodents quickly and reliably learn the predictive nature of the CS and US; that is, the CS predicts the US and the CS-alone comes to elicit conditioned behavioral and autonomic fear responses (CRs) such as freezing behavior, increased heart rate, and respiration. Importantly, these CRs can be reduced by presentation of the CS in the absence of the US, a process termed extinction which results in a new CS-no US memory. In this review, we aim to dissect the existing literature on the role for LC-NE in mediating the effects of acute stress on Pavlovian fear conditioning and extinction in animals. We posit that aversive stimuli (“stressors”, such as footshock) increase arousal by driving LC-NE release in the forebrain. In the present review, we operationally define “arousal” as the heightened noradrenergic tone that follows an aversive event. We suggest that NE dynamically modulates Pavlovian conditioning and extinction, either promoting or impairing learning aversive processes under different levels of behavioral arousal.

## The LC-NE System Anatomy and Physiology

The LC is a bilateral brainstem nucleus located adjacent to the fourth ventricle. While the LC is small in terms of cell count (~1500 in rats and ~15,000 in humans) it has been implicated in a range of behavioral phenomenon (Aston-Jones and Cohen, 2005; Arnsten, 2009, 2015; Robbins and Arnsten, 2009; Sara, 2009, 2015; Aston-Jones and Waterhouse, 2016). The LC was first described in detail by Dahlstrom and Fuxe in 1964 (Dahlström and Fuxe, 1964). This discovery led to a surge of interest into this small nucleus in the subsequent decades. Below we review the broad afferent and efferent connectivity of the LC and briefly describe the different receptor subtypes of the NE system.

### Efferents

The LC projects broadly throughout the brain and is largely thought to be the sole source of cortical NE. Due to its vast projections, it is not surprising that NE has an important role in many aspects of behavior and cognition. LC neurons fire at low basal rates (~1–3 Hz) and can fire in two modes: phasic states of firing (i.e., bursts of activity) occur in response to relevant environmental stimuli whereas the LC fires tonically during periods of stress. Increased tonic firing rates are associated with less phasic activity (Aston-Jones et al., 1999; Aston-Jones and Cohen, 2005). How these distinct firing modes affect aversive learning and memory is not well characterized. The LC consists of at least two cell types with the smaller fusiform cells being found in more dorsal portions of the nucleus whereas larger multipolar cells tend to be located more ventrally (Swanson, 1976; Grzanna and Molliver, 1980). Because this review is largely focused on fear conditioning and extinction, we restrict our focus to projections to the medial prefrontal cortex (mPFC), the basolateral amygdala (BLA, encompassing all nuclei), the central amygdala (CeA) and the hippocampus (HPC) all of which are heavily innervated by the LC (Descarries and Lapierre, 1973; Lapierre et al., 1973; Pickel et al., 1974; Segal and Landis, 1974; Swanson and Hartman, 1975; Swanson, 1976; Amaral and Sinnamon, 1977; Descarries et al., 1977; Jones and Moore, 1977; Fallon et al., 1978; Gerfen and Clavier, 1979; Moore and Bloom, 1979; Morrison et al., 1979; Loughlin et al., 1982; Foote et al., 1983). Recent work has led to an increasingly complex view of LC function based on the discovery of target-specific subpopulations within this small nucleus. We will discuss the contribution of distinct LC efferents in more detail throughout the review as it relates to aversive learning and memory.

### Homogeneous or Heterogeneous Output?

Upon the initial discovery of the LC it was determined that all neurons within this nucleus were noradrenergic (Dahlström and Fuxe, 1964). Further anatomical work on the extensive projections of the LC promoted the idea that this nucleus was largely homogenous, serving to distribute NE throughout the forebrain to coordinate global brain states. For example, several tracing studies describe collateralization of LC projections (Swanson and Hartman, 1975; Nagai et al., 1981; Room et al., 1981; Steindler, 1981; Jones and Yang, 1985). In addition, physiological evidence supported this idea–LC firing properties were found to be topographically homogeneous, phasic activity was synchronized amongst neurons, and local field potentials also displayed high synchronization (Aston-Jones and Bloom, 1981a; Ishimatsu and Williams, 1996). How could the LC-NE system dynamically modulate so many different aspects of behavior and cognition which depend on distinct brain regions/systems if its effects are global, rather than task and target specific?

Indeed, others have argued that the LC is comprised of distinct target-specific subpopulations. Several tracing studies have shown the LC consists of largely non-overlapping populations of neurons that can be defined based on their downstream target (Loughlin et al., 1982, 1986; Waterhouse et al., 1983; Chandler and Waterhouse, 2012; Agster et al., 2013; Chandler et al., 2013, 2014; Uematsu et al., 2015, 2017; Waterhouse and Chandler, 2016; Hirschberg et al., 2017). Waterhouse and colleagues have provided extensive anatomical and physiological evidence to suggest that the LC does not simply distribute NE equally to its many targets. For example, in a series of studies using retrograde tracers infused into different cortical regions, they have shown strong evidence for separate populations of cells within the LC (Waterhouse et al., 1983; Chandler and Waterhouse, 2012; Agster et al., 2013; Chandler et al., 2013, 2014; Waterhouse and Chandler, 2016). Moreover, they have recorded from these distinct LC populations and demonstrated that LC neurons projecting to the mPFC (compared to motor cortex) show different molecular properties that promote increased excitability. The cellular properties of target-specific LC populations may be related to the functional needs of their unique downstream targets (Chandler et al., 2014; Waterhouse and Chandler, 2016). A recent study has further confirmed the LC has highly divergent projections (i.e., it is not completely homogeneous) and suggests that small subpopulations may be selective for target regions, but propose that the LC may still serve to dictate brain-wide states (Schwarz and Luo, 2015; Schwarz et al., 2015). Moving forward, it will be important to examine the target specificity of the LC as well as how phasic vs. tonic firing in these discrete populations affect their downstream target to influence learning and memory.

### Synaptic or Volume Transmission?

A second issue regarding how the LC influences both brain-wide states, as well as distinct target regions, revolves around the potential mechanism of NE transmission. How the LC releases NE has been an area of debate with two possible mechanisms receiving attention. Traditional synaptic release of NE being one possibility and the other being volume transmission, or nonsynaptic release. Some evidence suggests that LC terminals release NE at traditional synapses (Papadopoulos et al., 1987, 1989; Papadopoulos and Parnavelas, 1990). In contrast, others have proposed that LC-NE functions primarily via volume transmission (i.e., nonsynaptic or extrasynaptic release; Descarries and Lapierre, 1973; Lapierre et al., 1973; Descarries et al., 1977; Agster et al., 2013). It is likely that the LC-NE system supports both synaptic and nonsynaptic release and this may be area specific (Olschowka et al., 1981; Farb et al., 2010). It is possible that volume transmission preferentially influences brain-wide states/NE-tone whereas synaptic release is dependent upon local needs of specific target regions, though these ideas remain to be tested.

### Afferents

Complementing its widespread projections throughout the forebrain, the LC receives dense reciprocal feedback from many of it’s targets. Indeed the LC is highly interconnected with the mPFC, BLA, CeA and HPC (Cedarbaum and Aghajanian, 1978; Arnsten and Goldman-Rakic, 1984; Aston-Jones et al., 1986; Sara and Hervé-Minvielle, 1995; Jodo et al., 1998; Van Bockstaele et al., 1998; Valentino and Van Bockstaele, 2008; Schwarz and Luo, 2015; Schwarz et al., 2015). The LC expresses several peptides including, but not limited to, vasopressin, somatostatin, neuropeptide y, enkephalin, neurotensin, corticotropin releasing hormone, galanin, glutamate, acetycholine and serotonin (Berridge and Waterhouse, 2003; Aston-Jones et al., 2004; Schwarz and Luo, 2015; Schwarz et al., 2015). These observations suggest that the LC is highly responsive to numerous transmitter and peptide systems and likely integrates information from several incoming sources. A recent study has shown that the LC receives direct projections from 111 brain regions (Schwarz et al., 2015). How the LC integrates this information is a subject of great interest. The LC has extensive dendritic arborization extending into the periocoerulear region which receives widespread non-NE synaptic contact (Shipley et al., 1996) and nearby GABAergic cells within this dendritic zone likely serve to regulate LC function (Aston-Jones et al., 2004). Understanding how the LC reciprocal network affects both LC signaling and target regions remains an important question.

### Receptor Subtypes

NE exerts its function via action at three G-protein coupled receptor subtypes with the α2-adrenoceptors (ARs; A, B and C subtypes) having the highest affinity, followed by α1-ARs (A, B and D), and the lowest affinity β-ARs (1, 2 and 3; Berridge and Waterhouse, 2003; Ramos and Arnsten, 2007). The heterogeneous distribution, distinct subtypes, and differing affinities of each class of receptor provide yet another mechanism by which NE may exert target-specific effects. The α2-ARs are G_i_-coupled leading to the inhibition of cAMP and thereby reducing neuronal excitability and primarily serve as presynaptic autoreceptors, although they are also expressed postsynaptically (MacDonald et al., 1997; Ramos et al., 2006; Ramos and Arnsten, 2007). Several studies have demonstrated strong expression patterns in the mPFC, HPC and amygdala using *in situ* hybridization (Zeng and Lynch, 1991; McCune et al., 1993; Nicholas et al., 1993b; Scheinin et al., 1994; Wang et al., 1996), radioligand binding (Unnerstall et al., 1984; Boyajian et al., 1987), and immunohistochemical techniques (Aoki et al., 1994; Rosin et al., 1996; Talley et al., 1996).

The α1-ARs are generally thought to be excitatory in nature and are G_q_-coupled. Activation of these receptors acts via phospholipase C and phosphatidyl inositol intracellular signaling mechanisms, activating protein kinase C and subsequent release of intracellular calcium (Johnson and Minneman, 1985; Marshall et al., 1999; Birnbaum et al., 2004; Ramos and Arnsten, 2007). This class of ARs can also be found throughout the cortex, HPC, and amygdala (Young and Kuhar, 1980; Rainbow and Biegon, 1983; Jones et al., 1985; Palacios et al., 1987; McCune et al., 1993; Pieribone et al., 1994; Day et al., 1997; Domyancic and Morilak, 1997); however, α2-ARs tend to be more widespread than α1-ARs (McCune et al., 1993). This may serve as a mechanism for target regions to regulate NE action to reduce signaling by having densely expressed, high-affinity autoreceptors.

Lastly, the lowest-affinity β-ARs are G_s_-coupled to adenylyl cyclase resulting in increased cAMP and enhanced cellular excitability (Ordway et al., 1987; Ferry et al., 1999a,b; Zhang H.-T. et al., 2005). β-ARs show high expression levels throughout the brain, particularly in the HPC, mPFC, and amygdala (Rainbow et al., 1984; Booze et al., 1993; Nicholas et al., 1993a; Summers et al., 1995; Milner et al., 2000). Interestingly, β-ARs are also expressed on astrocytes which may indirectly influence neural signaling (Milner et al., 2000). Signaling via α1- and β-ARs has been proposed to have opposing effects on the mPFC and BLA. High levels of NE may bias instinctive and reflexive responses mediated by NE action at α1- and β-ARs in the BLA and whereas activation of these receptors may impair mPFC function. This has important implications for aversive learning and memory (Arnsten, 2009, 2015; Arnsten et al., 2015).

## Stress, the LC-NE System and the Fear Circuit

The LC responds to both appetitive and aversive stimuli (Aston-Jones and Bloom, 1981b; Sara and Segal, 1991; Aston-Jones et al., 1999; Bouret and Sara, 2004; Aston-Jones and Cohen, 2005; Ventura et al., 2008; Aston-Jones and Waterhouse, 2016), however the focus of this section will be to examine how NE affects key nodes in the fear circuit. Footshock serves as the US in the majority of Pavlovian fear conditioning experiments, and it is well document that footshock and other acute stressors increase LC activity (Thierry et al., 1968; Sara and Segal, 1991; Smith et al., 1992; Pezzone et al., 1993; Passerin et al., 2000; Sved et al., 2002; Chen and Sara, 2007; George et al., 2013; Uematsu et al., 2017). Below we discuss how LC activity and NE affects the fear circuit.

### NE and the Amygdala

The BLA plays a crucial role in the formation and retrieval of fear conditioning and extinction memories (LeDoux, 2000; Maren, 2001, 2011; Maren and Quirk, 2004; Myers and Davis, 2007; Johansen et al., 2011; Herry and Johansen, 2014; Dejean et al., 2015). NE signaling in the amygdala appears to be critical for most aspects of Pavlovian fear conditioning and extinction (see below). Increased LC activity in response to acute stressors (including footshock) produces robust increases in amygdalar NE content (Galvez et al., 1996; Quirarte et al., 1998; McGaugh, 2000, 2004; Morilak et al., 2005; Ramos and Arnsten, 2007; Arnsten, 2009, 2015). How increased NE affects BLA signaling is therefore a fundamental question when studying emotional learning and memory. It has been proposed that heightened NE levels in the amygdala promote instinctive and reflexive responses to environmental stimuli (which would presumably bias responses for emotional events and memories); (Southwick et al., 1999a; Ramos and Arnsten, 2007; Arnsten, 2009, 2015; Arnsten et al., 2015). For example, one study found that footshock-induced increases in LC and BLA Fos were significantly reduced by LC inhibition prior to footshock (using the GABA_A_ antagonist muscimol). Moreover, drugs that increase NE efflux (such as the α2 autoreceptor antagonist yohimbine) produce robust increases in BLA Fos expression (Singewald et al., 2003). This suggests that heightened noradrenergic activity in the BLA promotes excitability which would likely strengthen fear memories. However, a pair of studies has demonstrated that footshock, LC stimulation, or iontophoresis of NE (or NE-increasing drugs) into the BLA produced heterogeneous responses in BLA single-unit activity, although the BLA was generally suppressed in response to increased NE (Buffalari and Grace, 2007; Chen and Sara, 2007). This is perhaps counterintuitive if increased amygdalar excitability is associated with fear memory formation and recall, but NE did increase the spontaneous firing rate of a smaller subpopulation of BLA neurons (Buffalari and Grace, 2007). Interestingly, the suppression of BLA firing is dependent upon α2-AR signaling insofar as iontophoresis of clonidine mimicked the effects of NE and these inhibitory effects are potentiated with the systemic administration of propranolol (Buffalari and Grace, 2007). It is possible that the smaller population of BLA cells that showed excitation is sufficient for memory formation or that higher levels of NE (that engage the lower affinity receptors) would result in more excitation.

The central amygdala (CeA) is viewed as the output region of the amygdala that drives fear expression, although mounting data indicate it too plays a role in the acquisition of fear conditioning (Goosens and Maren, 2003; Yu et al., 2017). That said, CeA microcircuits and projections to downstream targets such as the periaqueductal gray are particularly important for generating freezing behavior (Ciocchi et al., 2010; Haubensak et al., 2010; Fadok et al., 2017). Importantly, the CeA is reciprocally connected with the LC (Van Bockstaele et al., 1998; Valentino and Van Bockstaele, 2008). Under stress, the CeA activates the LC via corticotropin-releasing hormone (Van Bockstaele et al., 1998; McCall et al., 2015; Prouty et al., 2017) which may act as a positive feedforward mechanism to maintain high levels of LC activity and NE transmission, particularly in the amygdala. This circuit could provide a way to generate sustained fear responses, particularly in the aftermath of conditioning.

### NE and the Hippocampus

The HPC is critical for integrating and processing spatial information which is important in context fear conditioning among other types of learning and memory (Bouton et al., 2006; Maren et al., 2013; Jin and Maren, 2015; Chen et al., 2017; Hansen, 2017) and LC input to the HPC has been shown to impact learning about a novel context (Wagatsuma et al., 2018). Indeed, NE has a major influence on hippocampal function and LC stimulation, footshock, and other acute stressors increase hippocampal NE levels (Abercrombie et al., 1988; Hajós-Korcsok et al., 2003; Yavich et al., 2005). In addition, drugs that increase NE levels, such as yohimbine, amplify this effect whereas drugs that reduce NE levels, such as clonidine, blunt stress-induced hippocampal NE release (Abercrombie et al., 1988). Moreover, a number of studies suggest that NE enhances hippocampal long-term potentiation (LTP), particularly in the dentate gyrus and CA1, which is dependent upon both α1 and β-AR mechanisms (Bliss et al., 1983; Neuman and Harley, 1983; Lacaille and Harley, 1985; Stanton and Sarvey, 1985a,b, 1987; Hopkins and Johnston, 1988; Dahl and Sarvey, 1989; Segal et al., 1991; Dunwiddie et al., 1992; Katsuki et al., 1997; Chaulk and Harley, 1998; Izumi and Zorumski, 1999; Yang et al., 2002; Harley, 2007). For example, NE applied either directly to the dentate gyrus or applied to the perforant pathway increases excitatory postsynaptic potentials, decreases spike onset latency, and increases the population spike amplitude; these effects promote LTP induction and may be important for memory formation (Neuman and Harley, 1983; Lacaille and Harley, 1985; Harley, 2007). However, NE effects on LTP may depend on stimulation parameters and the areas being stimulated which suggests that NE can dynamically modulate HPC function (Dahl and Sarvey, 1989; Harley, 2007). In line with this idea, restraint stress and tail shock (which would presumably increase hippocampal NE) have been shown to impair hippocampal LTP (Foy et al., 1987). It may be that stress-induced increases in HPC-NE are beyond optimal levels and exceed the levels used in many of the recording studies showing that NE enhances HPC-LTP. Despite these possibilities, it appears NE generally enhances hippocampal synaptic efficacy which may function to enhance emotional learning and memory. Of course, this may be sensitive to the prevailing level of NE and the subregions being examined, allowing NE to bidirectionally modulate HPC function.

### NE and the mPFC

The prelimbic (PL) and infralimbic (IL) subdivisions of the mPFC are thought to regulate the expression and suppression of fear, respectively (Quirk and Mueller, 2008; Knapska and Maren, 2009; Herry et al., 2010; Milad and Quirk, 2012; Dejean et al., 2015; Giustino and Maren, 2015). Several studies have examined the effects of NE and stress on prefrontal function. Footshock and other acute stressors increase NE levels in the mPFC (Korf et al., 1973; Gresch et al., 1994; Finlay et al., 1995; Hatfield et al., 1999; Ishizuka et al., 2000; Morilak et al., 2005; Girotti et al., 2017). Similar to other brain regions, NE effects on PFC function are highly dependent upon the prevailing level of NE and the task requirements. Lower levels of NE (engaging postsynaptic α2-ARs) appear to promote cortical function such as cognitive flexibility and working memory, whereas high levels impair prefrontal signaling via α1- and β-AR dependent mechanisms (Arnsten and Li, 2005; Ramos et al., 2006; Ramos and Arnsten, 2007; Arnsten, 2009, 2015). Interestingly, the PFC has more dopamine-β hydroxylase (DβH) varicosities relative to sensory cortical areas (Agster et al., 2013). This raises the possibility that prefrontal regions might be subjected to greater release of NE (via volume transmission in addition to synaptic transmission), which may help explain why the PFC is highly sensitive to stress. An important topic for future research will be to address differences in LC projections to PL and IL and how these affect fear expression.

## NE and Fear Conditioning

As mentioned above the mPFC, HPC and BLA are a triad of interconnected brain regions with important roles in Pavlovian fear conditioning. Here we will review direct and indirect manipulations of LC-NE on both cued and context fear conditioning and the neural circuit mechanisms underlying these effects.

### Cued Fear Conditioning

Several studies reveal that LC-NE plays an essential role in the acquisition of cued fear conditioning and this may be dependent upon projections to the BLA (Oei and King, 1980; Goldstein et al., 1996; Pitman and Delahanty, 2005; Liu et al., 2007; Tully et al., 2007; Rodrigues et al., 2009; Tully and Bolshakov, 2010; Johnson et al., 2011; Krugers et al., 2011; Pitman et al., 2012; Uematsu et al., 2017). This is likely due to stress-induced LC activation in response to the footshock US. However, a trained CS can also elicit increased LC activity. For example, early investigations demonstrated that c-fos mRNA is increased in the LC after presentation of a CS and that 6-hydroxydopamine (6-OHDA) lesions of NE (particularly the dorsal noradrenergic bundle, DNAB) impair fear conditioning to explicit cues (Cole and Robbins, 1987; Selden et al., 1990; Smith et al., 1992). However, some studies found no effects of 6-OHDA lesions (Mason and Fibiger, 1979) while others saw enhanced aversion with DNAB lesions (Selden et al., 1991). These differences may relate to the extent of damage.

Indirect manipulations of LC-NE (i.e., genetic and/or pharmacological approaches targeting NE transmission) lend support to the idea that NE is a critical factor for the acquisition of cued fear conditioning. For example, there is extensive evidence to suggest that β-ARs are important for fear acquisition insofar as systemic or intra-BLA propranolol (non-selective β1,2 antagonist) prior to training impairs cued fear conditioning (Cole and Koob, 1988; Bush et al., 2010; Díaz-Mataix et al., 2017; Schiff et al., 2017). Interestingly, methylphenidate (increases NE/DA levels by blocking NET and DAT) enhanced cued fear memory formation at lower levels, but had an impairing effect at higher doses (Carmack et al., 2014). In addition, blunting NE transmission with the α2-AR agonist dexmedetomidine resulted in weakened cued conditioning; this was associated with decreased Fos expression in both the BLA and CeA (Davies et al., 2004). In line with this, intra-BLA infusions of clonidine also impaired fear acquisition (Schulz et al., 2002). Curiously, α1 blockade (via terazosin), either systemically or intra-BLA, actually enhanced fear memory formation (Lazzaro et al., 2010). The authors found that terazosin facilitated LTP in the lateral amygdala which would support fear learning (Lazzaro et al., 2010). These findings suggest that NE can have opposing and dynamic effects on the acquisition of fear learning depending on the circuits being investigated and the subtype of AR being manipulated.

Genetic manipulations also suggest that NE signaling primarily promotes fear learning. Mutations that result in reduced NE transmission blunt, whereas those that increase NE signaling enhance conditioning (Kim et al., 1997; Kobayashi and Kobayashi, 2001; Davies et al., 2003). However, mice completely lacking NE or epinephrine appear to acquire cued fear normally (Murchison et al., 2004, 2011; Ouyang and Thomas, 2005; Toth et al., 2013). Several of these studies were done with DBH knockout (KO) mice using a single conditioning trial. While the interpretation of these studies would suggest that NE is not necessary for most aspects of fear conditioning (both cued and contextual) a recent study may help explain these paradoxical results. NE enhances fear learning for multi-trial, but not single-trial, conditioning protocols (Díaz-Mataix et al., 2017). It may be that weaker conditioning protocols, such as single-trial conditioning paradigms, do not engage the LC-NE system to the same extent as more typical, multi-trial training protocols. This will be noted where necessary throughout the review because the results from several DBH KO studies have led to a view that NE may play little to no role in many aspects of cued and context fear that were examined in a series of studies using weak conditioning protocols that may not rely on NE transmission (Murchison et al., 2004; Ouyang and Thomas, 2005; Zhang W.-P. et al., 2005; Schutsky et al., 2011a,b). It would be of great interest to know if these mice show deficits in most aspects of aversive learning and memory when trained with multiple trials.

**Figure 1:**
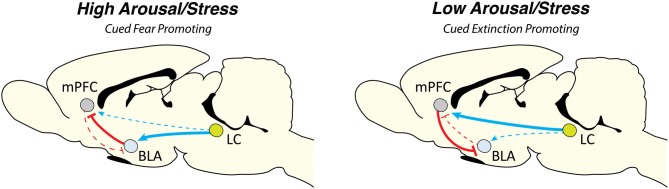
Locus coeruleus norepinephrine (LC-NE) dynamically regulates cued aversive learning and memory. We propose a model by which the LC-NE system can dynamically regulate cued Pavlovian fear conditioning and extinction based on the prevailing level of stress at the onset of learning. Under high levels of arousal the LC-NE system acts via α1 and β-ARs, a state that would favor elevated levels of fear and hinder new learning (for example, in the case of immediate extinction). High levels of stress may act to increase BLA function while simultaneously impairing mPFC output which is necessary for extinction learning. In contrast, under low levels of arousal, LC-NE acts via α2-ARs in both the BLA and mPFC. This scenario would likely promote new learning (particularly extinction learning) by leaving mPFC function intact. Arrow width indicates projection strength. Abbreviations: mPFC, medial prefrontal cortex; BLA, basolateral complex of the amygdala; LC, locus coeruleus.

Fanselow and colleagues have developed a stress-enhanced fear learning paradigm in which they have shown that pre exposure to unsignaled shocks enhances fear conditioning to both contexts and cues (Rau et al., 2005; Rau and Fanselow, 2009). It would be interesting to examine the role of the LC and NE in modulating these effects. We have recently shown that the mPFC is particularly sensitive to footshock stress. Both the PL and IL showed rapid and in some cases sustained alterations in spontaneous firing rate. In particular, IL activity was suppressed for nearly an hour following cued fear conditioning (which corresponded with high levels of freezing) and these effects were mitigated by systemic propranolol (Fitzgerald et al., 2015). A recent study has shown that LC projections to the BLA, but not the mPFC, underlie the acquisition of cued fear memories (Uematsu et al., 2017). Overall, the existing data suggest that NE strongly affects the acquisition of cued fear conditioning and these effects may be due to enhanced BLA activity as well as impaired mPFC function under high levels of stress associated with conditioning (see Figure 1).

### Contextual Fear Conditioning

Norepinephrine also influences the acquisition of contextual fear conditioning, although these effects may be due to the aforementioned effects on hippocampal LTP in addition to effects on the BLA. Evidence suggests LC-NE may be a critical mediator of contextual fear learning insofar as LC neurons (and GABAergic neurons near/within the LC) show increased Fos expression following context fear conditioning (Ishida et al., 2002). In addition, 6-OHDA lesions of the LC result in impaired contextual fear conditioning (Neophytou et al., 2001). These data suggest that footshock stress strongly activate the LC-NE system to promote contextual fear learning. However, others have reported that either 6-OHDA or DNAB (but not VNAB) lesions actually enhance context fear (Selden et al., 1990, 1991). It is possible the conflicting results stem from differences in the extent of LC-NE damage.

Genetic and pharmacological manipulations of NE largely support the idea that NE regulates context fear learning. For instance, genetic or pharmacological depletion of NE results in impaired contextual fear (Onaka et al., 1996; Murchison et al., 2004, 2011; Ouyang and Thomas, 2005; Zhang W.-P. et al., 2005; Hott et al., 2012) and this corresponded with a reduction in CA1 activation (Zhang W.-P. et al., 2005). Moreover, increasing levels of NE or epinephrine promotes context fear learning (Kim et al., 1997; Frankland et al., 2004; Hu et al., 2007; Carmack et al., 2014). Somewhat paradoxically, increasing NE levels with dexmedetomidine or via α2-AR KO seemingly had no effects on context fear (Davies et al., 2003, 2004), although a recent study has demonstrated that intra-CeA clonidine blocks the acquisition of context fear (Holmes et al., 2017). These discrepancies may relate to differing levels of NE with each manipulation (i.e., differences in the conditioning protocols), species differences, and/or compensatory mechanisms in mutant mice.

The HPC richly expresses low affinity β-ARs and their activation has a demonstrated role in enhanced HPC-LTP (Harley, 2007). It seems likely that HPC-LTP is highly sensitive to the level of NE efflux which may explain how NE can bidirectionally modulate context fear acquisition, by either enhancing or impairing LTP. Connectivity between the HPC and mPFC may importantly mediate effects on context learning, which has been shown to be affected by NE in the BLA. Recent work suggests that NE in the BLA bidirectionally affects HPC-mPFC plasticity. NE enhanced HPC-mPFC LTP in an α2-AR dependent fashion and β-AR stimulation decreased LTP in this pathway (Lim et al., 2017). These data provide compelling evidence that the BLA may gate HPC-mPFC plasticity which may promote high and low fear states in a receptor-dependent manner. How this affects contextual fear learning remains an open question.

## NE and Fear Expression

### Cued Fear Expression

In line with the idea that NE is important for the acquisition of both cued and contextual fear conditioning, it appears that NE also modulates fear expression. As mentioned above, a trained CS has been demonstrated to engage the LC-NE system and CS-exposure produces a conditional fear response associated with noradrenergic arousal. In terms of cued fear expression, systemic administration of propranolol prior to extinction training reduced within session freezing which corresponded with a reduction in PL activity (Rodriguez-Romaguera et al., 2009). Similarly, decreasing NE transmission with clonidine produced dose-dependent reductions in fear expression whereas elevating NE (piperoxane or yohimbine) increased fear expression as measured by potentiated startle in the presence of a formerly trained CS (Davis et al., 1979). This finding was replicated with intra-LA infusions of clonidine (Schulz et al., 2002). These effects may depend upon interactions between the BLA and mPFC insofar as post-conditioning lesions of the BLA blocked CS-induced increases in mPFC NE levels which correlated with lower fear expression (Goldstein et al., 1996). Moreover, slice recordings have shown that while NE did not disrupt glutamatergic BLA signaling, inhibitory neurotransmission was decreased in the BLA following recall of a cued fear memory. These data suggest that NE and heightened fear expression is associated with BLA excitability (Skelly et al., 2017). Interestingly, a handful of reports suggest NE manipulations had no effect on cued fear expression (Murchison et al., 2004, 2011; Ouyang and Thomas, 2005; Schutsky et al., 2011b; Laitman et al., 2014). As discussed above, these findings come from studies with DBH KO mice. Importantly, the training protocols used in these studies may help explain the observed effects (or lack thereof). These studies used a single shock conditioning trial and a recent study has demonstrated that NE blockade has no effects on single-trial conditioning, but is necessary for multi-trial enhancement of learning and subsequent expression of that memory (Díaz-Mataix et al., 2017). Weaker conditioning protocols are apparently less likely to recruit LC-NE given that they appear insensitive to NE manipulations.

### Contextual Fear Expression

Similar to the effects reported on the acquisition of context fear, NE manipulations impact context fear expression. For instance, genetic depletion of NE produces deficits in context fear retrieval and this effect is dependent upon β-AR signaling insofar as these deficits are recapitulated by propranolol (Murchison et al., 2004, 2011; Ouyang and Thomas, 2005; Zhang W.-P. et al., 2005). Interestingly, xamoterol (β1 partial agonist) actually impairs context freezing in a dose-dependent fashion; however, the authors argue that these effects are mediated by G_i_-coupled β2 signaling which acts to oppose G_s_-coupled β1 signaling which is necessary for hippocampal-dependent memory retrieval (Schutsky et al., 2011a,b). Moreover, pharmacologically driven increases in NE (acute treatment with reobxetine, a norepinephrine reuptake inhibitor) enhanced context fear expression (Inoue et al., 2006). It would be interesting to know if DBH KO mice show similar deficits using a multi-trial conditioning protocol which would more likely to result in stress-induced LC-NE signaling.

## NE and Memory Consolidation

After initial training occurs short-term memory undergoes consolidation processes to form a long-term memory. Post-training manipulations have been used to examined the involvement of NE in both cued and context fear memory consolidation. An interesting dichotomy emerges here, where it seems that NE is not necessarily needed for the consolidation of cued fear learning, but is for context.

### Cued Fear Consolidation

While NE (particularly in the BLA) is critical for the formation of cued fear memories it may not be essential for the consolidation of such memories. Mice lacking NE showed no deficits in cued fear memory consolidation using single-trial conditioning procedures (Murchison et al., 2004; Ouyang and Thomas, 2005). Further supporting this idea, post training systemic administration of either epinephrine or amphetamine had no effects on consolidation (Lee et al., 2001). Moreover, systemic, intra-mPFC, or intra-BLA propranolol has no impact on fear memory consolidation (Bush et al., 2010; Fitzgerald et al., 2015; Giustino et al., 2017; Schiff et al., 2017). and post training propranolol had no effects on LA synaptic strengthening (Clem and Huganir, 2013). In line with this, intra-BLA atenolol only impaired corticosterone enhanced cued fear memories, but had no effect in rats that just received auditory fear conditioning and post-training BLA infusions of atenolol (Roozendaal et al., 2006). Interestingly, mice heterozygous for a mutation in the gene encoding tyrosine hydroxylase (a precursor enzyme in the biosynthetic pathway for NE) show impaired cued fear memory and this could be restored by drug-induced NE stimulation after training (Kobayashi et al., 2000; Kobayashi and Kobayashi, 2001). However, these mutant mice only show a modest reduction in NE accumulation and release; they may also have differences in the distribution and density of AR expression as a compensatory mechanism complicating the interpretation of the results. The existing literature primarily suggests that NE manipulations have minimal consequences for the consolidation of cued fear conditioning memories.

### Contextual Fear Consolidation

As noted above, an intriguing difference emerges when comparing the role of NE in cued vs. contextual fear memory consolidation. This may relate to the extensive and long-lasting effects of NE manipulations on hippocampal LTP, which would favor the strengthening of contextual memories. Increasing NE with yohimbine administration has been shown to enhance context fear memory consolidation. This enhancement was blocked with propranolol administration, suggesting a role for β-ARs. In addition, dampening NE-transmission with clonidine reduced contextual fear memory consolidation (Gazarini et al., 2013, 2014). These effects seem to be dependent on both the BLA and HPC insofar as post training NE into the BLA enhances consolidation (McGaugh and Roozendaal, 2002; LaLumiere et al., 2003) and blockade of β-ARs in CA1 has impairing effects (Ji et al., 2003). These findings lend support to the idea that NE importantly regulates the consolidation of HPC-dependent contextual fear memories in a β-ARs dependent manner. The involvement of NE in the consolidation of contextual, but not cued, fear conditioning suggests that NE is operating on the consolidation of context representations, rather than context-US or CS-US associations. Moreover, signaled and unsignaled shocks produced marked differences in neuronal activity in the mPFC (Fitzgerald et al., 2015; Giustino et al., 2016a), which may play a role in contextual memory (Zelikowsky et al., 2013, 2014; Sharpe and Killcross, 2014, 2015; Heroux et al., 2017; Pennington et al., 2017).

## NE and Reconsolidation

When a memory is reactivated it enters a labile state and undergoes protein synthesis dependent reconsolidation (Nader et al., 2000; Duvarci et al., 2008). This provides a window of opportunity to disrupt long-term memories. We and others have previously reviewed the effects of NE (mainly examining the effects of propranolol) on reconsolidation and will therefore only briefly touch on this area in this review (Steckler and Risbrough, 2012; Giustino et al., 2016b; Kroes et al., 2016a). Disrupting the reconsolidation of a fear memory can potentially have far reaching clinical implications for trauma- and stressor-related disorders. Many labs have provided compelling evidence that propranolol delivered after brief memory reactivation disrupts the reconsolidation of both cued and context fear memories in rodents (Przybyslawski et al., 1999; Dębiec and Ledoux, 2004; Abrari et al., 2008; Muravieva and Alberini, 2010; Dębiec et al., 2011; Pitman et al., 2011; Schneider et al., 2014; Taherian et al., 2014). In addition, systemic clonidine has also mirrored these effects (Gamache et al., 2012). An important consideration regarding these findings is that reconsolidation effects may be subject to certain boundary conditions such as the age and strength of a memory, thus making interference with fear memories more complicated (Duvarci et al., 2006; Wang et al., 2009). Moreover, many of these conditioning protocols use a single-trial, producing a relatively weak fear memory that is less dependent upon NE transmission (Díaz-Mataix et al., 2017), which may be more susceptible to blockade.

## NE and Fear Extinction

Behavioral and pharmacological approaches aimed at improving extinction learning in rodents have received much attention due to the clinical importance of extinction based cognitive behavioral therapies (Myers and Davis, 2007; Quirk and Mueller, 2008; Holmes and Quirk, 2010; Mueller and Cahill, 2010; Vervliet et al., 2013; Fitzgerald et al., 2014; Giustino and Maren, 2015; Giustino et al., 2016b). It is generally thought that stress impairs extinction learning and this may relate to alterations in NE transmission (Mason and Fibiger, 1979; Mason et al., 1979; Holmes and Quirk, 2010; Steckler and Risbrough, 2012; Fitzgerald et al., 2015; Giustino et al., 2016b, 2017; Maren and Holmes, 2016).

### Cued Fear Extinction

While NE primarily strengthens the acquisition of fear learning, it has bidirectional effects on extinction learning that may depend on the extant level of noradrenergic arousal. For example, reducing or depleting NE levels impairs delayed extinction (occurring at least 24 h following fear conditioning; Mason and Fibiger, 1979; Tsaltas et al., 1984; Cole and Robbins, 1987; Cain et al., 2004; Mueller et al., 2008; Fitzgerald et al., 2015) and this may be due to effects on IL activity (Mueller et al., 2008). In line with this, recent work has shown that LC projections to the mPFC promote extinction learning (Uematsu et al., 2017). Indeed increasing NE with yohimbine can facilitate delayed extinction learning (Cain et al., 2004; Morris and Bouton, 2007; Hefner et al., 2008; Mueller et al., 2009). These findings suggest that NE is important for the formation of a new and competing CS-no US extinction memory.

Our lab and others have demonstrated that the timing of extinction training relative to conditioning is an important factor regulating the long-term success of extinction in rodent models and humans (Maren and Chang, 2006; Chang et al., 2010; Kim et al., 2010; MacPherson et al., 2013; Stafford et al., 2013; Maren, 2014; Fitzgerald et al., 2015; Hollis et al., 2016; Merz et al., 2016; Giustino et al., 2017). These effects may be due to high levels of stress (and NE) soon after conditioning that interferes with new learning. In line with this idea, we have shown that systemic or intra-BLA propranolol enables learning where it normally fails (Fitzgerald et al., 2015; Giustino et al., 2016b, 2017). In addition, systemic propranolol administered prior to delayed extinction, when stress levels are presumably lower, impaired learning when tested in a drug free state the following day (Fitzgerald et al., 2015). These data are supported by the idea that stress generally impairs extinction learning which may be due to alterations in NE signaling (Lin et al., 2016; Maren and Holmes, 2016). Importantly, the fact that reducing NE signaling prior to delayed extinction has impairing effects suggests that CS-evoked NE actually facilitates learning under lower levels of arousal. In the case of immediate extinction (a state of higher arousal at the onset of learning), it is likely that propranolol is reducing NE signaling to more optimal levels, thus facilitating learning. Our data and others suggest that heightened NE in the BLA strengthens fear memories perhaps at the expense of forming a new extinction memory. Overall, NE can bidirectionally modulate extinction learning and this seems to depend on the prevailing level of stress at the onset of learning. Low levels of NE can enhance prefrontal function which may act to blunt downstream signaling in the BLA to promote extinction learning (Figure 1). These findings have important clinical implications and warrant careful consideration when interpreting effects of NE-altering drugs and their effects on the treatment of trauma- and stressor-related disorders (discussed further below).

### Contextual Fear Extinction

As with context fear, the extinction of contextual fear may rely heavily on hippocampal function. It seems that NE is critical to context fear extinction (and its consolidation). Methylphenidate (a NET and DAT blocker) delivered either before or after context extinction enhanced learning in a dose-dependent manner (Abraham et al., 2012). These effects may be due to increased synaptic efficacy in the HPC and BLA insofar as NE infusion into CA1 or the BLA immediately after context extinction mirror these enhancing effects (Berlau and McGaugh, 2006; Chai et al., 2014). In addition, NE in the vmPFC has been implicated in enhanced context extinction learning such that β-AR agonism (via isoproterenol) facilitates learning whereas systemic propranolol or prazosin hinder learning (Cain et al., 2004; Bernardi and Lattal, 2010; Do-Monte et al., 2010). These data support the notion that NE is a critical factor for the extinction of contextual fear. It is likely that these effects are at least in part mediated by HPC plasticity, which can be regulated by NE transmission. As discussed above, recent work has shown that NE in the BLA can alter HPC-mPFC plasticity (Lim et al., 2017). Given the important role of the mPFC in extinction learning and evidence that NE levels that engage α2-AR signaling enhance HPC-mPFC plasticity, it remains possible that this may be a neural mechanism underlying contextual fear extinction.

## NE and PTSD

Noradrenergic modulating drugs are used to treat an array of neuropsychiatric disorders, though the only two FDA approved drugs for PTSD are selective serotonin reuptake inhibitors (Steckler and Risbrough, 2012; Tawa and Murphy, 2013; Fitzgerald et al., 2014; Arnsten et al., 2015). Drugs that either elevate or reduce NE transmission have been studied and used off-label for the treatment of PTSD and its symptoms with varying success (Southwick et al., 1997, 1999a,b,c; Holmes and Quirk, 2010; Bukalo et al., 2014; Giustino et al., 2016b). It has recently been shown that threat is associated with increased LC activity in healthy human volunteers and this acts to strengthen prioritized memory representations (Clewett et al., 2018). This suggests that heightened states of arousal may promote fear memory formation and maintenance. In addition, a number of studies have demonstrated that elevated NE plays a major role in the pathophysiology of PTSD (Kosten et al., 1987; Yehuda et al., 1992; Bremner et al., 1996; Southwick et al., 1997, 1999a,b,c; Geracioti et al., 2001; Strawn and Geracioti, 2008; Naegeli et al., 2018). Despite this link, success with pharmacological erasure of fear memories and/or enhancement of extinction based cognitive behavioral therapies has been limited (Bos et al., 2014).

It has been proposed that individuals with PTSD may “hypercondition” to fearful stimuli and this is coupled with impaired extinction, ultimately resulting in a heavy bias towards fearful responses in inappropriate situations (Orr et al., 2000; Lissek et al., 2005; Guthrie and Bryant, 2006; Blechert et al., 2007; Wessa and Flor, 2007; Milad et al., 2008, 2009; Pitman et al., 2012; VanElzakker et al., 2014; Norrholm et al., 2015). While the LC-NE system plays an important role in learning and memory, including extinction learning (Sterpenich et al., 2006; Ramos and Arnsten, 2007; Arnsten, 2009, 2015; Sara, 2009, 2015; Arnsten et al., 2015), stress (and elevated NE beyond optimal levels) may only exacerbate these effects by impairing extinction learning and/or increasing generalization (Hartley et al., 2014; Raio et al., 2014, 2017; Raio and Phelps, 2015; Maren and Holmes, 2016; Dunsmoor et al., 2017). Indeed, yohimbine has been used in healthy human subjects to enhance fear learning and it also has been shown to hinder extinction learning (van Stegeren et al., 2010; Soeter and Kindt, 2011b, 2012; Visser et al., 2015). Despite this, there has been some interest in yohimbine as a pharmaceutical agent to augment the treatment of PTSD, which has yielded mixed results (Powers et al., 2009; Holmes and Quirk, 2010; Wangelin et al., 2013).

Clonidine and guanfacine are two α2-AR agonists that are used to treat a number of conditions. While the evidence for the efficacy of either drug is somewhat limited, these drugs may have some use in the treatment of PTSD and related disorders (Arnsten et al., 2015; Belkin and Schwartz, 2015). These NE-reducing agents have been shown to reduce symptoms of hyperarousal associated with PTSD as well as sleep disturbances (Kinzie and Leung, 1989; Horrigan and Barnhill, 1996; Porter and Bell, 1999; Boehnlein and Kinzie, 2007; Detweiler et al., 2016). Importantly, both compounds have also shown safety and promise for treating PTSD in children (Harmon and Riggs, 1996; Connor et al., 2013). Unfortunately, some studies have found little evidence for the efficacy of α2-AR agonists in the treatment of PTSD (Neylan et al., 2006; Davis et al., 2008). It remains possible that these, or related drugs, may only be effective for individuals who have dysregulated/elevated NE signaling which may explain discrepant findings. Further research is warranted on the efficacy of these compounds.

Results with noradrenergic receptor antagonists, such as propranolol, have yielded mixed results in both healthy human volunteers and individuals with PTSD. Propranolol is already used safely in humans for other conditions and has been shown to reduce long-term memory for an emotionally arousing story (Cahill et al., 1994) and reduced the strength of context conditioning in healthy human subjects (Grillon et al., 2004). These data suggest that β-AR activation underlies memories for emotional events. However, some have suggested that propranolol treatment soon after trauma has no effects, although this was done in the absence of any extinction based therapy (Stein et al., 2007; McGhee et al., 2009; Nugent et al., 2010). In addition, one report found that propranolol has no effects on the acquisition or retention of extinction learning (Orr et al., 2006). Others have shown that propranolol impairs extinction learning in healthy subjects (Bos et al., 2012). At first glance, these conflicting reports would imply that researchers and clinicians alike should look elsewhere for a pharmaceutical adjunct for exposure therapy. However, we have argued that the timing of propranolol administration coupled with behavioral therapy is an often overlooked, and highly important, factor regulating the long-term outcome of extinction learning (Giustino et al., 2016b). This may be due to differences in the prevailing level of noradrenergic arousal at the time of administration. There is some empirical evidence to support this idea in humans though more research coupling propranolol and extinction soon after trauma is needed (Pitman et al., 2002; Vaiva et al., 2003).

One area that has shown promise centers on blocking the reconsolidation of a fearful memory. Several studies in both healthy human volunteers and individuals with PTSD have suggested that propranolol can be used to disrupt reconsolidation (Brunet et al., 2008, 2011, 2014; Kindt et al., 2009; Soeter and Kindt, 2011a, 2012; Poundja et al., 2012; Schwabe et al., 2012; Lonergan et al., 2013). As discussed previously, effects on reconsolidation may be subject to certain boundary conditions and memories do not necessarily even undergo reconsolidation unless new learning occurs (Sevenster et al., 2012). Moreover, many of these reconsolidation effects have not been replicated which further complicates approaches focusing on reconsolidation blockade as an effective treatment strategy for PTSD (Tollenaar et al., 2009; Bos et al., 2014; Spring et al., 2015; Wood et al., 2015). It seems unlikely that acute administration of propranolol, or any drug, would effectively eradicate a long-standing fear memory, such as those observed in individuals suffering from PTSD. However, this does not preclude the idea that propranolol may reduce fear under some circumstances and thus has utility moving forward (Giustino et al., 2016b; Kroes et al., 2016a,b).

Another noradrenergic receptor antagonist that has been used primarily to combat disordered sleep in individuals with PTSD is the α1-AR antagonist prazosin. Prazosin has shown promise in ameliorating nightmares and sleep disturbances associated with PTSD (Raskind et al., 2003; Taylor et al., 2008; Koola et al., 2014; Writer et al., 2014; de Dassel et al., 2017; Keeshin et al., 2017; Miller et al., 2017; Short et al., 2018). However, a recent clinical trial demonstrated that prazosin did not ameliorate sleep-related disturbances in military veterans with PTSD (Raskind et al., 2018). Less is known about how prazosin affects other aspects of PTSD symptomatology. A recent study in healthy human subjects suggests that prazosin delivered prior to fear conditioning enhanced future discrimination between fearful and safe stimuli during extinction (Homan et al., 2017). Further work is needed to examine the effects of prazosin as well as other NE-altering drugs in both healthy human volunteers and those with PTSD.

## Conclusion

Overall, the LC-NE system critically regulates most aspects of emotional learning and memory in rodent models, healthy human subjects, and individuals suffering from trauma- and stressor-related disorders. Recent advances in technology for basic science research will be crucial to further our understanding of how stress and the LC-NE system regulate these effects in rodent models. Target-specific approaches have led to a new appreciation of LC function and while the precise effects of distinct LC subpopulations are not well characterized several recent articles have pinpointed unique contributions to learning and memory as well as anxiety in rodents (Schwarz and Luo, 2015; Schwarz et al., 2015; Uematsu et al., 2015, 2017; Li et al., 2016; Hirschberg et al., 2017; McCall et al., 2017).

An important area of research moving forward may center around individualized medicine based on differences in the LC-NE system and stress responsivity. Human imaging protocols have improved to better isolate the LC (Keren et al., 2009; Murphy et al., 2014; Betts et al., 2017; Brooks et al., 2017; Krebs et al., 2017; Langley et al., 2017; Priovoulos et al., 2017; Song et al., 2017; Tona et al., 2017). Understanding if and how LC-NE is contributing to an individual’s symptomatology will likely improve therapeutic outcomes. Patients often undergo several “rounds” of drug treatment as they (and their doctor) search for either a single or combination of agents that ameliorate their condition. An improved appreciation of how the LC-NE system contributes to aversive learning and memory and its subsequent extinction may help improve empirically driven treatment options.

We propose that the LC-NE system dynamically regulates the acquisition and extinction of both cued and contextual fear and these effects are dependent upon the prevailing level of stress (and NE) at the onset of learning (Figure 1). LC-NE likely influences learning and memory processes in a manner described by an inverted-U function, albeit acting in different brain regions/circuits to regulate cued and contextual learning. Regarding contextual fear conditioning, we suggest that the LC-NE system acts to regulate hippocampal LTP, which is involved in the acquisition, consolidation, and extinction of contextual representations (as opposed to context-US associations). In terms of cued fear conditioning, low levels of NE release prior to delayed extinction would enhance extinction learning by promoting mPFC function, which would, in turn, inhibit BLA output to enable extinction learning. In contrast, high levels of NE released under stress (such as that accompanying footshock) promotes fear expression while inhibiting new learning (as is observed with immediate extinction procedures) by strengthening BLA function and simultaneously impairing mPFC function via target-specific LC subpopulations.

## Author Contributions

TFG and SM wrote and edited the manuscript.

## Conflict of Interest Statement

The authors declare that the research was conducted in the absence of any commercial or financial relationships that could be construed as a potential conflict of interest.
